# Iron homeostasis in *Bacillus subtilis* relies on three differentially expressed efflux systems

**DOI:** 10.1099/mic.0.001289

**Published:** 2023-01-17

**Authors:** Caroline H. Steingard, Azul Pinochet-Barros, Brian M. Wendel, John D. Helmann

**Affiliations:** ^1^​ Department of Microbiology, Cornell University, Ithaca, New York 14853-8101, USA

**Keywords:** *Bacillus subtilis*, cation diffusion facilitator, iron, manganese, metal homeostasis, regulation, oxidative stress

## Abstract

In *Bacillus subtilis,* iron homeostasis is maintained by the ferric uptake regulator (Fur) and manganese homeostasis relies on the manganese transport regulator (MntR). Both Fur and MntR function as bi-functional metalloregulators that repress import and activate metal ion efflux systems. The ferrous iron efflux ATPase, PfeT, is derepressed by hydrogen peroxide (H_2_O_2_) as sensed by PerR and induced by iron as sensed by Fur. Mutants lacking PfeT are sensitive to iron intoxication. Here, we show that *mntR* mutants are also iron-sensitive, largely due to decreased expression of the MntR-activated MneP and MneS cation diffusion facilitator (CDF) proteins previously defined for their role in Mn^2+^ export. The ability of MneP and MneS to export iron is apparent even when their expression is not induced by Mn^2+^. Our results demonstrate that PfeT, MneP and MneS each contribute to iron homeostasis, and a triple mutant lacking all three is more iron-sensitive than any single mutant. We further show that sensitivity to H_2_O_2_ does not correlate with iron sensitivity. For example, an *mntR* mutant is H_2_O_2_-sensitive due to elevated Mn(II) that increases PerR-mediated repression of peroxide resistance genes, and this repression is antagonized by elevated Fe^2+^ in an *mntR pfeT* mutant. Thus, H_2_O_2_-sensitivity reflects the relative levels of Mn^2+^ and Fe^2+^ as sensed by the PerR regulatory protein. These results underscore the complex interplay between manganese, iron and oxidative stress in *B. subtilis *.

## Introduction

In *Bacillus subtilis *, iron and manganese homeostasis are intimately intertwined through the coordinated action of three metalloregulatory proteins: the Fe^2+^ sensor Fur, the Mn^2+^ sensor MntR and the oxidative stress response regulator PerR, which functions with either Fe^2+^ or Mn^2+^ [1–4]. The ferric uptake repressor Fur is the primary iron sensing metalloregulator and represses the transcription of genes encoding iron acquisition proteins under iron replete conditions [5–7]. Iron acquisition is highly redundant, with systems for uptake of several different siderophores, elemental iron, and iron citrate [6]. Under iron-limiting conditions, Fur also indirectly represses expression of abundant iron-containing enzymes such as succinate dehydrogenase and glutamate synthase through the small RNA FsrA, which represses translation [8, 9]. Finally, under iron-overload conditions, Fur is a direct transcriptional activator of the P_1B4_-type ATPase PfeT [10], which serves as an Fe^2+^ efflux pump [11–13].

Manganese (Mn^2+^) is also an essential micronutrient in *B. subtilis* [1, 14]. Mn^2+^ homeostasis is controlled by MntR, a homodimeric protein with an N-terminal DNA-binding domain and a C-terminal dimerization domain [15, 16]. Each subunit binds to two Mn^2+^ ions to activate DNA binding [17]. Strains lacking *mntR* are highly sensitive to Mn^2+^ intoxication [15]. This can be attributed, in part, to derepression of Mn^2+^ import mediated by MntH and an ABC transporter, MntABC [15, 18]. In addition, MntR activates transcription of two cation diffusion facilitator (CDF) Mn^2+^ efflux pumps, MneP and MneS [19].

The adaptive response to hydrogen peroxide (H_2_O_2_) stress is coordinated by the Fur paralogue, PerR [5, 20]. PerR binds either Fe^2+^ or Mn^2+^ at its metal-sensing site, which triggers the allosteric transition needed for high affinity DNA binding [21–23]. In response to H_2_O_2_, iron-cofactored PerR is inactivated by oxidation of an iron-coordinating histidine residue [22, 24], and the inactive protein is then degraded [25]. This regulatory mechanism is conserved amongst PerR orthologues [26], although the sensitivity to oxidative inactivation varies and is tuned to the ambient levels of Fe^2+^ and Mn^2+^ in the cytosol of different organisms [27, 28]. The PerR regulon comprises genes that encode enzymes to directly detoxify H_2_O_2_ (catalase KatA and alkyl hydroperoxide reductase AhpCF) and proteins that alleviate toxicity by affecting iron levels [29–31]. The latter proteins include MrgA (a mini-ferritin that sequesters iron), haem biosynthesis enzymes (required for KatA function), Fur and the iron efflux pump PfeT [11, 32]. Because *pfeT* is under dual regulation by PerR and Fur, iron efflux is elevated in response to either excess intracellular iron or peroxide stress [10, 11]. Orthologues of PfeT are similarly regulated in several Gram-positive pathogens and are known to be important for virulence in the host [33–37].

Here, we demonstrate that iron homeostasis reflects the combined action of three efflux systems with overlapping metal selectivity (PfeT, MneP, MneS). PfeT functions as an iron-activated efflux system critical under iron overload conditions, whereas MneP and MneS have iron efflux activity even at their basal level of expression. Cells with decreased capacity for iron efflux have increased iron sensitivity but are not necessarily more sensitive to H_2_O_2_. This is consistent with the ability of PerR to repress peroxide resistance genes with either Mn^2+^ or Fe^2+^ as co-repressor, but to sense H_2_O_2_ only in its Fe^2+^-cofactored form.

## Methods

### Bacterial strains, phage, plasmids and growth conditions

*B. subtilis* strains derived from CU1065 (WT) were grown on lysogeny broth (LB) medium (10 g l^−1^ casein digest peptone, 5 g l^−1^ yeast extract, 5 g l^−1^ NaCl) at 37^ ^°C with vigorous shaking. The strains used in this study are listed in Table S1, available in the online version of this article. Ferrous iron (FeSO_4_·7H_2_O) was titrated into the media as indicated from a freshly made 100 mM iron stock solution dissolved in 1 N HCl unless otherwise indicated. SPβ phage are derivatives of SPβ*c*2Δ2 and were constructed by integration of a promoter region*–cat–lacZ* operon fusion constructed in pJPM122 into strain ZB307A as described previously [38]. Ampicillin (amp; 100 μg ml^−1^) was used to select *E. coli* transformants. Erythromycin (ery; 1 μg ml^−1^) and lincomycin [linc; 25 μg ml^−1^; for testing macrolide–lincosamide–streptogramin B (MLS) resistance], spectinomycin (spec; 100 μg ml^−1^), kanamycin (kan; 10 μg ml^−1^) and neomycin (neo; 10 μg ml^−1^) were used for the selection of various *B. subtilis* strains.

### Overexpression strain construction

For complementation, PCR products were amplified from genomic DNA, digested with HindIII and BglII, and cloned into pPL82 [39]. All oligonucleotides in this study are listed in Table S2. The resulting constructs, which allow IPTG-inducible expression of genes, were linearized with PstI and integrated into the *B. subtilis* chromosome at the *amyE* locus.

### Reporter strain construction

The P*_pfeT_–cat–lacZ* operon fusion was generated in strain ZB307A [38] and moved to different backgrounds by SPβ transduction and selection for MLS and neomycin resistance as described elsewhere [10, 38]. Strains were confirmed by PCR.

### Zone of inhibition (ZOI) assays

Strains were grown to an OD_600_ of 0.4. A 100 µl aliquot of these cultures was mixed with 4 ml of 0.75 % LB soft agar (kept at 50 °C) and directly poured onto LB plates (containing 15 ml of 1.5 % LB agar). The plates were dried for 10 min in a laminar airflow hood. Filter paper discs containing 5 µl (streptonigrin, SN) or 10 µl of the chemicals to be tested were placed on the top of the agar, and the plates were incubated at 37 °C overnight. The overall diameter of the inhibition zones was measured along two orthogonal lines. For IPTG-treated cells, IPTG was added to both the soft agar and the plates to a concentration of 0.1 mM. For media containing additional MnCl_2_ or FeSO_4_, metal solutions were added to both the soft agar and the plates to a final concentration of 15 µM and 100 µM, respectively. Unless otherwise noted, 10 µl of the following chemicals was used in the disc diffusion assays: 1 M FeSO_4_ and 0.88 M H_2_O_2_. For SN sensitivity tests, FeSO_4_ was added to both the soft agar and the plates to a concentration of 0.1 mM and 5 µl of 5 mg ml^−1^ SN solution in dimethyl sulfoxide (DMSO) was added to the filter paper discs.

### ImageJ zone of lower density analysis

Photographs of the zone of inhibition plates were taken with a Nikon D5000 camera and converted to black and white images. A pixel density histogram across a highlighted region was generated using ImageJ.

### β-galactosidase assays

Cells containing the *pfeT* promoter *lacZ* fusions (P_pfeT_–*lacZ*) were grown in LB amended with different concentrations of FeSO_4_·7H_2_O in milliQ H_2_O to an OD_600_ of ~0.4 in 96-well plates with 200 µl LB medium per well at 37 °C with vigorous shaking. Cells were pelleted by centrifugation, resuspended in Z buffer supplemented with dithiothreitol (DTT, 400 nM final concentration) and lysed by lysozyme. OD_600_ was measured before lysozyme treatment. After lysis, ortho-nitrophenyl-β-galactoside (ONPG) was added and OD_420_ and OD_550_ were measured every 2 min. Product accumulation was calculated using the formula product=1000×[OD_420_−(1.75×OD_550_)] and plotted against time. The slope of the linear part of the product accumulation curve was calculated using Excel and Miller unit (MU) was calculated using the formula MU=slope/OD_600_/*V*, where *V* is the volume of cells used for the reaction (200 µl).

### Quantification of intracellular metal content by inductively coupled plasma mass spectrometry (ICP-MS)

Cells were grown in LB medium overnight and subcultured with 1 : 100 ratio into LB medium to an OD_600_ of ~0.4. Cells were harvested, and levels of intracellular Fe were monitored by ICP-MS. All samples were washed once with chelex-treated phosphate-buffered saline (PBS) buffer containing 1 mM EDTA and then twice with chelex-treated PBS. Cell pellets were resuspended in 400 µl of buffer 2 (1× chelex-treated PBS buffer, 75 mM NaN_3_, 1 % Triton X-100) and incubated at 37 °C for 90 min to lyse the cells. Lysed samples were spun down by centrifugation and the total protein content was quantified using a Bradford assay. Then, samples were mixed with 600 µl buffer 4 [5 % HNO_3_, 0.1 % (v/v) Triton X-100] and heated in a 95 °C sand bath for 30 min. The debris was removed by centrifugation and the total metal ions in the diluted samples were analysed by Perkin-Elmer ELAN DRC II ICP-MS. Gallium was used as an internal standard. (mean±se; *n*=3).

### qRT-PCR

Strains of interest were grown in LB medium to an OD_600_ of ~0.5 and 1.5 ml of culture was used for RNA extraction. RNA isolation (Qiagen, USA) and cDNA preparation (Thermo Fisher, USA) was carried out as suggested by the manufacturer. qRT-PCR was carried out using a Bio-Rad iTaq universal SYBR green super mix. 23S rRNA was used to normalize the cycle threshold (*C*_t_) value.

## Results

### An *mntR* mutant is sensitive to iron intoxication

**Fig. 1.**
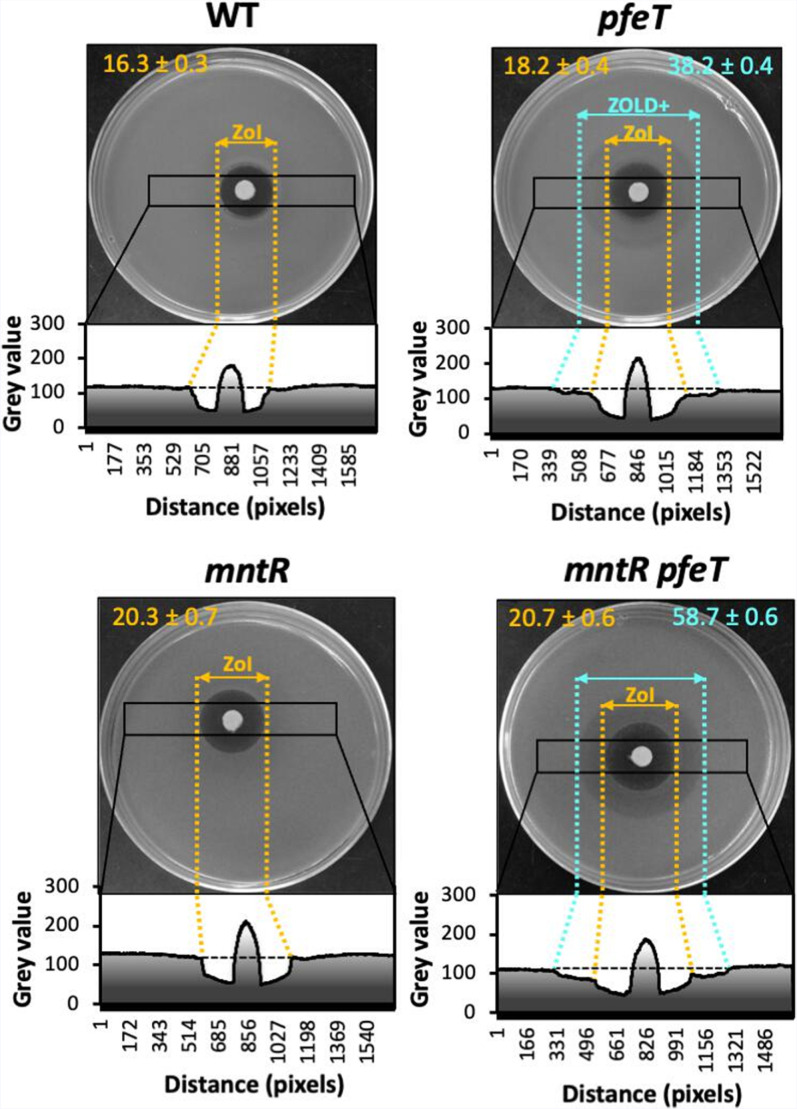
Densitometry analysis of Fe disc diffusion assay. Representative images of the iron disc diffusion assay plates were converted to black and white images. The inner zone of inhibition (ZOI) is surrounded by a zone of lower density (ZOLD). The ZOI is labelled in orange and the additional diameter due to the ZOI+ZOLD (ZOLD+) is labelled in blue. Values shown are the mean and standard deviation (*n*=3). A black rectangle on images denotes the area selected to perform ImageJ densitometry analysis. Respective densitometry histograms are shown below each strain image. A dashed black line is shown to highlight the difference between the basal pixel density of the cell lawn and the less dense areas of the ZOI (extending to dotted orange line) and ZOLD (extending to dotted blue line).

**Fig. 2.**
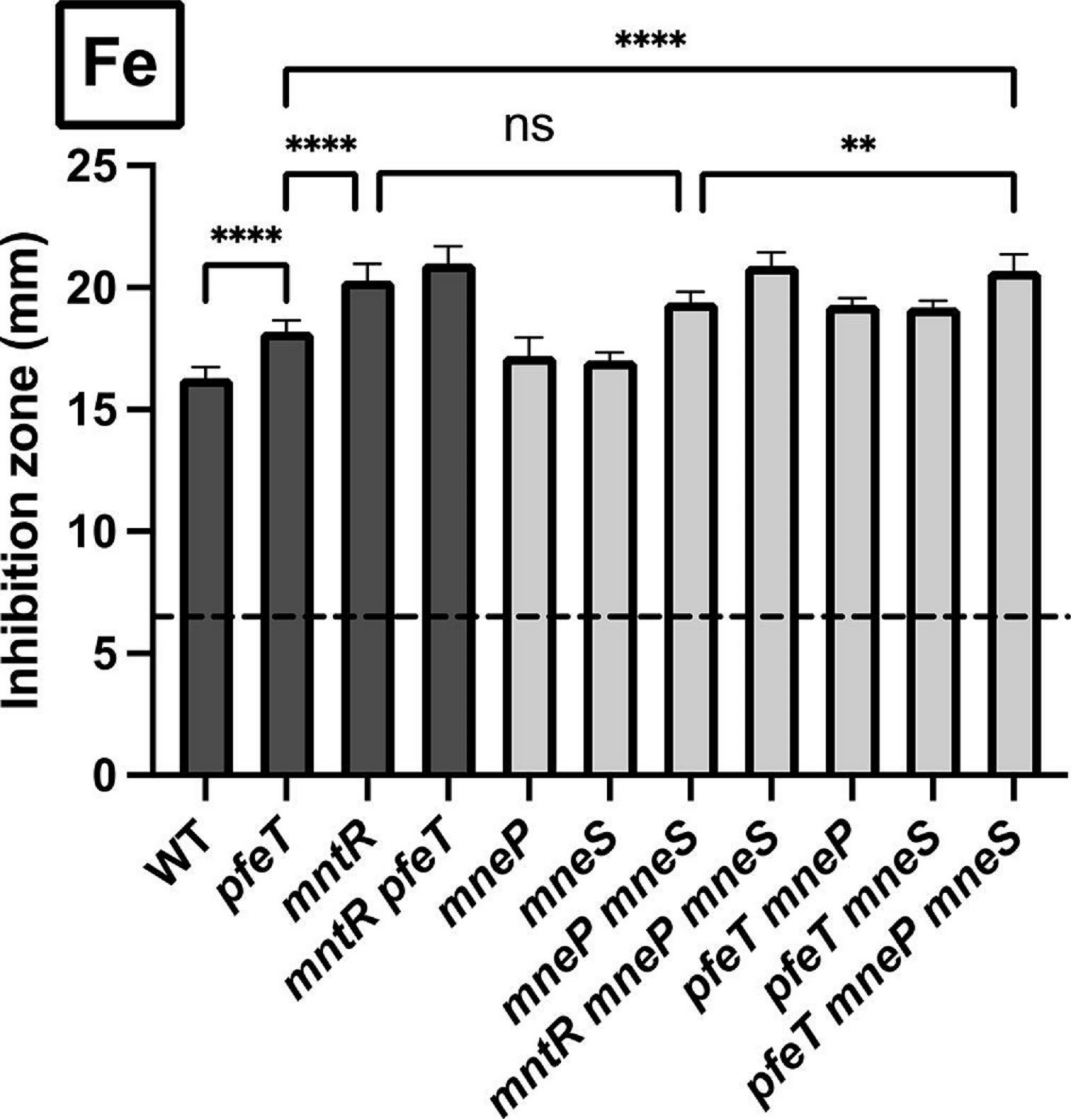
The MntR regulon impacts iron homeostasis via MneP and MneS. Sensitivity of WT and mutants to Fe^2+^ as monitored using a disc diffusion (ZOI) assays. Bars in dark grey show the strains from Fig. 1, whereas light grey bars represent mutants affected in metal efflux. The black dashed line indicates the diameter of the disc (6.5 mm). Values are ZOI diameter (mm) and are the mean±standard deviation (sd) for *n*=5. A one-way analysis of variance (ANOVA) test was performed followed by a Tukey test for multiple comparisons with corrections (**, <0.02; ****, <0.0001), ns indicates not significant

A *B. subtilis pfeT* mutant is sensitive to iron overload as monitored by growth inhibition in a disc diffusion assay [11]. Iron intoxication is revealed by an inner ZOI, as well as an adjacent zone of lower density (ZOLD) growth, which is absent in wild-type (WT) cells, as judged using Image J (Fig. 1). Previously, we observed that iron intoxication of *pfeT* mutants was strongly ameliorated by the addition of micromolar levels of Mn^2+^ [11]. This suggests that iron intoxication may be due to the inhibition of one or more Mn^2+^-dependent enzymes by Fe^2+^ mismetalation. Instead of supplementing the medium with Mn^2+^, we hypothesized that *mntR* null mutants, which constitutively express Mn^2+^ import proteins, might also suppress the iron sensitivity of the *pfeT* mutant. Contrary to this expectation, the *mntR pfeT* double mutant had an even greater iron sensitivity than the *pfeT* mutant, and even the *mntR* single mutant was iron-sensitive (Figs 1 and 2). This result led us to hypothesize that one or more members of the MntR regulon affect iron homeostasis.

### MntR confers resistance to iron intoxication through MneP and MneS

MntR is both a repressor of Mn^2+^ import genes *mntH* and *mntABCD* and a transcriptional activator for the *mneP* and *mneS* efflux genes [19]. We used ZOI assays to determine whether the iron sensitivity of the *mntR* null mutant was due to increased metal import or reduced efflux. Iron sensitivity in the *mneP mneS* double mutant was comparable to the *mntR* single (Fig. 2). In contrast with Mn^2+^, a known inducer of *mneP* and *mneS* transcription, iron does not induce expression (Fig. S1). Previous results demonstrate that both *mneP* and *mneS* are expressed even in unamended LB medium, and this basal level of expression also requires activation by MntR [19]. We therefore hypothesized that the *mntR* mutant was iron sensitive due to a reduction in expression of the MneP and MneS efflux proteins.

The PfeT P_1B4_-type ATPase has been previously implicated as the major, iron-inducible mechanism for Fe^2+^ efflux [11]. We were therefore surprised to find that the *mneP mneS* double mutant was at least as iron-sensitive as a *pfeT* mutant (Fig. 2). However, the apparent Fe^2+^ affinity of PfeT is low, with a K_1/2_ of 520±120 µM for ATPase activation [11]. This suggests that the MneP and MneS cation diffusion facilitator (CDF) transporters may export Fe^2+^ and that this activity contributes to resistance even in cells containing PfeT. Moreover, the triple efflux mutant (*pfeT mneP mneS*) is more sensitive than either *pfeT* alone or the *mneP mneS* double mutant. We therefore hypothesize that PfeT, MneP, and MneS all contribute independently to Fe^2+^ efflux.

In an *mntR* mutant, Mn^2+^ import genes *mntH* and *mntABCD* are constitutively expressed. In *B. subtilis *, these are primarily involved in Mn^2+^ uptake, but homologous systems in other organisms have been shown to also import Fe^2+^ [40–44]. We therefore tested *mntR* mutant strains additionally lacking either *mntH* or *mntA* to monitor whether loss of either importer would restore tolerance to high iron. Both *mntR mntH* and *mntR mntA* strains are just as iron-sensitive as an *mntR* mutant (Fig. S2). In addition, deletion of *mntH* did not reduce the iron sensitivity of either *pfeT* or *mntR pfeT* mutant strains (Fig. S2). Thus, elevated expression of Mn^2+^ uptake genes in *mntR* mutant strains does not seem to play a major role in iron intoxication.

### Induction of MneP or MneS reduces iron intoxication

**Fig. 3.**
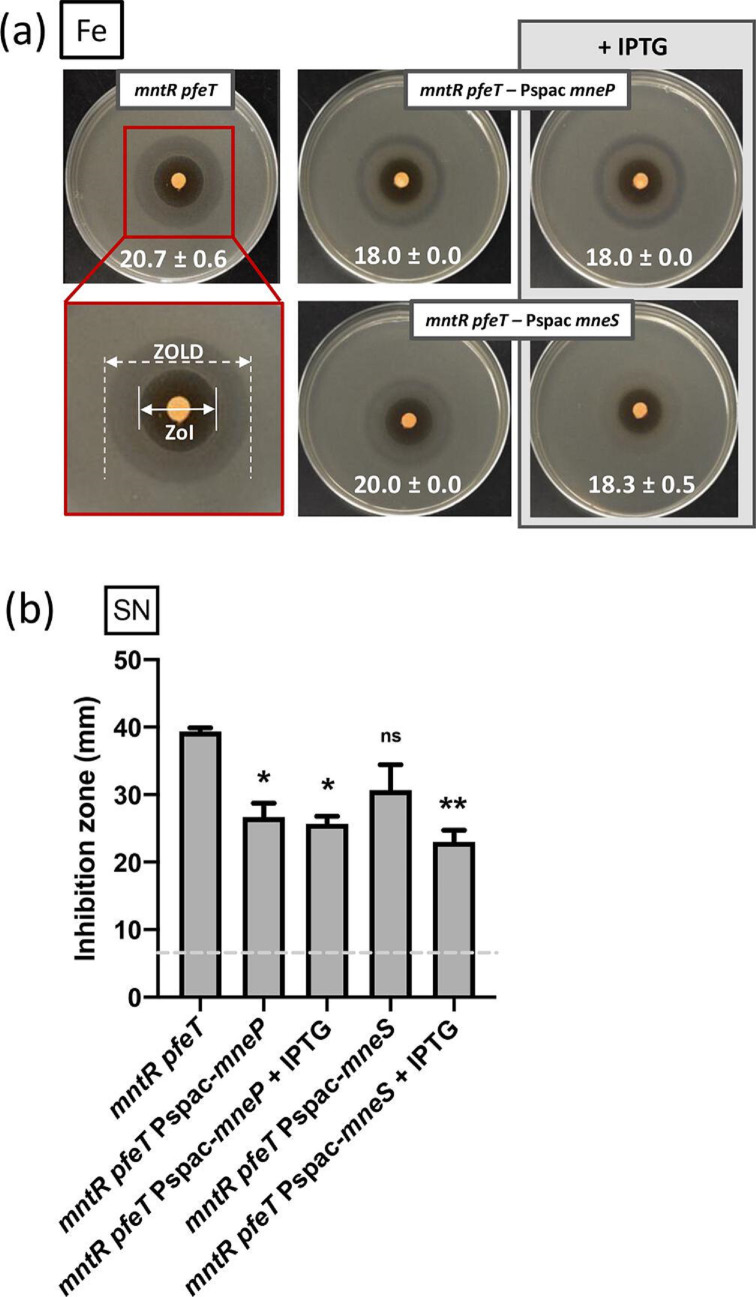
Induction of MneP or MneS reduces iron intoxication. (**a**) Representative images of ZOI plates with *mntR pfeT* strains showing ectopic expression of *mneP* and *mneS*. Inner ZOI values are the mean ± sd (*n*=3) and are shown at the bottom of each representative image. The red box on the *mntR pfeT* mutant image magnifies the ZOI region and the zone of lower density (ZOLD) on the plate for better comparison across strains. Light grey box shows addition of 1 mM IPTG added to the plate and the soft agar. (**b**) Streptonigrin (SN) ZOI assay in LB media with and without 100 µM Fe^2+^ in strains with an IPTG-inducible copy of *mneP* or *mneS*. Light grey line indicates diameter of the disc (6.5 mm). Values shown are the mean ± sd (*n*=3). A two-way ANOVA test with multiple comparison was performed with a Šidák correction (****, <0.0001; ns, no significance).

We used ectopic expression of *mneP* and *mneS* to test the hypothesis that the high iron sensitivity of the *mntR pfeT* double mutant is related to reduced expression of the MneP and MneS CDF proteins. Complementation with either gene reduced the iron sensitivity of the *mntR pfeT* double mutant to the level seen in a *pfeT* single mutant, as judged by the ZOI (Fig. 3a). These complementation strains were constructed with the pPL82 plasmid containing P_hyspac_ (denoted P_spac_ here), and leaky expression accounts for the increased resistance even in the absence of IPTG. Further, induction of *mneS* (but not *mneP*) almost completely suppressed the ZOLD in the *mntR pfeT* double mutant (Fig. 3a). These results hint that MneS may have a stronger impact than MneP on Fe^2+^ efflux under these conditions.

As an alternative approach to assess bioavailable iron we measured sensitivity to streptonigrin (SN), a quinone antibiotic with bactericidal activity that is indicative of intracellular iron availability [45]. To assess SN sensitivity, we used a ZOI assay on LB with and without 100 µM added Fe^2+^. In this assay, the *pfeT* single mutant displayed little change in ZOI (complete growth inhibition) but had a pronounced ZOLD, indicative of reduced fitness (Fig. S3), as shown previously [11]. The *mntR* single mutant had a larger ZOI than WT in both LB and LB plus 100 µM Fe^2+^, but did not display the ZOLD (Fig. S3). In the *mntR pfeT* double mutant the SN sensitivity seen in the single mutants was additive in the LB+100 µM Fe^2+^ medium. As a result, the *mntR pfeT* ZOI almost completely encompasses both the ZOI and ZOLD seen in a *pfeT* mutant (Fig. S3). We hypothesized that the introduction of the *mntR* mutation increased intracellular iron levels in this strain by decreasing expression of the MntR-activated MneP and MneS efflux pumps. Indeed, ectopic expression of either of these two efflux pumps partially restores SN resistance (Fig. 3b and Fig. S4). As expected, SN resistance of the *mntR pfeT* mutant is also complemented when PfeT is ectopically expressed (Fig. S3).

### Iron sensitivity in an *mntR* mutant is due to increased iron accumulation

**Fig. 4.**
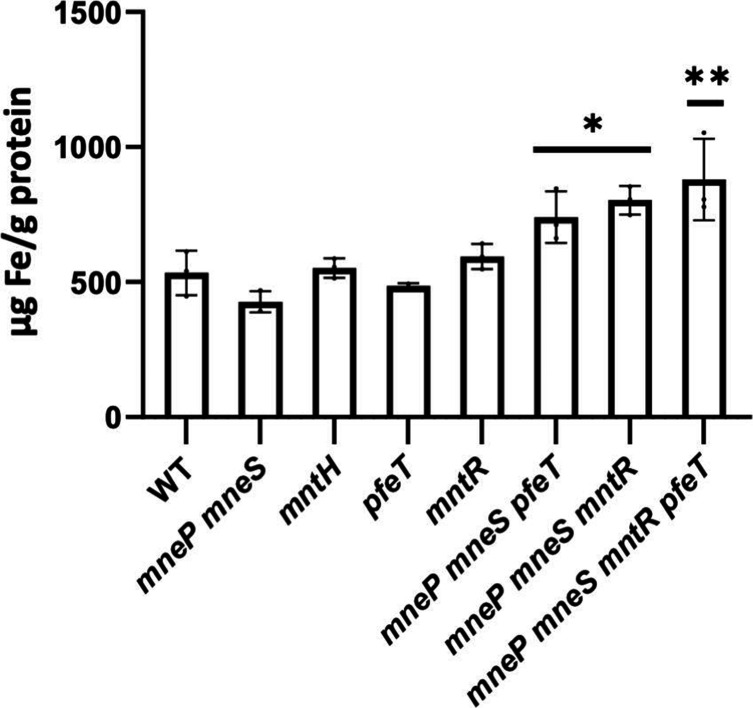
*mntR* and *mntR pfeT* mutants show iron accumulation via ICP-MS. Intracellular Fe content was measured in exponentially growing cells in LB medium by ICP-MS. Values shown are the mean ± sd (*n*=3). A one-way ANOVA test was performed. Asterisks represent comparison to the WT (*, <0.01, **, <0.001).

To directly assess how these mutations alter iron homeostasis, we measured total intracellular iron by ICP-MS analysis. For cells grown in non-stressed conditions (LB medium; ~13 µM iron as judged by ICP-MS), the total iron concentration within the *mneP mneS*, *mntR*, *pfeT* or *mntH* strains was not significantly different than WT (Fig. 4). However, the strain missing all three proteins implicated in iron efflux (*mneP mneS pfeT*) had a significant increase in intracellular iron. This supports the inference that all three proteins contribute independently to Fe^2+^ efflux, even in unamended LB medium. As shown previously, *B. subtilis* growing logarithmically in LB medium partially derepresses the Fur regulon to facilitate Fe^2+^ import, with full repression requiring amendment with an additional 25 µM iron [7]. Thus, intracellular iron homeostasis is a dynamic balance between import and export. Moreover, in unamended LB medium the mutation of *mntR* further increased intracellular iron levels in both the *mneP mneS* mutant and the triple efflux mutant (Fig. 4). This indicates that MntR-repressed metal importers (MntH and/or MntABCD) likely contribute to iron import under these conditions.

**Fig. 5.**
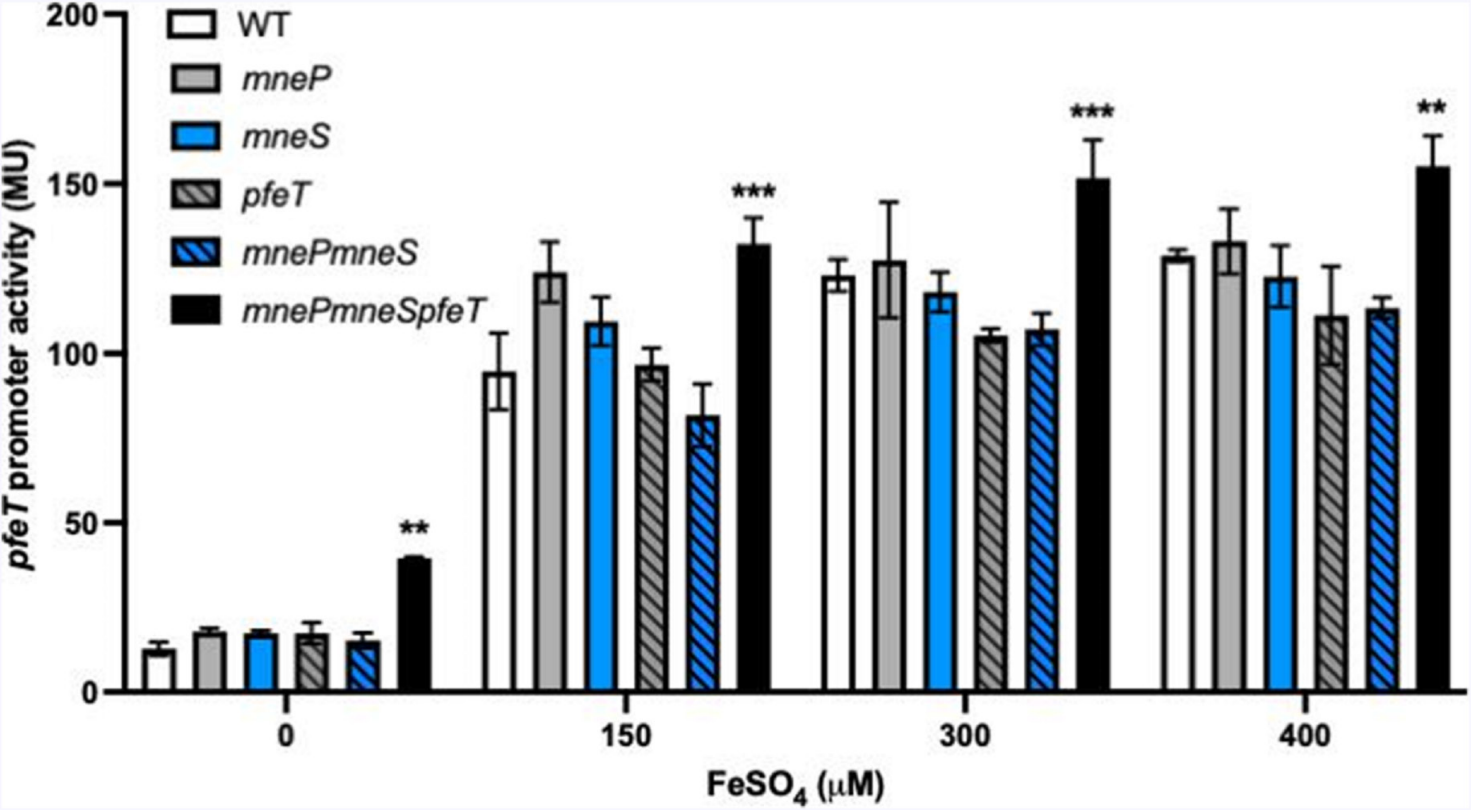
*mntR* and *mntR pfeT* mutants show iron accumulation via a *pfeT* transcriptional bioreporter. β-galactosidase assays to monitor P*_pfeT_–lacZ* expression in WT, *mneP, mneS, pfeT, mnePS* and *mnePS pfeT* mutant backgrounds grown in LB medium amended with different concentrations of FeSO_4_. Graph shows the mean ± sd (*n*=3). A one-way ANOVA test followed by a Tukey multiple comparison test was performed with corrections (**, <0.02; ***, <0.001).

While ICP-MS provides accurate measurements of total intracellular iron, only a subset of this metal pool is bioavailable. To assess bioavailable iron over a range of medium conditions, we introduced a Fur-activated P*_pfeT_*–lacZ transcriptional fusion into WT and various mutant backgrounds [10]. The *mneP mneS pfeT* triple efflux mutant displayed the highest expression in all conditions (Fig. 5). There was even elevated expression in unamended LB medium, consistent with the elevated levels of intracellular iron observed using ICP-MS (Fig. 4).

### Loss of *mntR* exacerbates H_2_O_2_ sensitivity

**Fig. 6.**
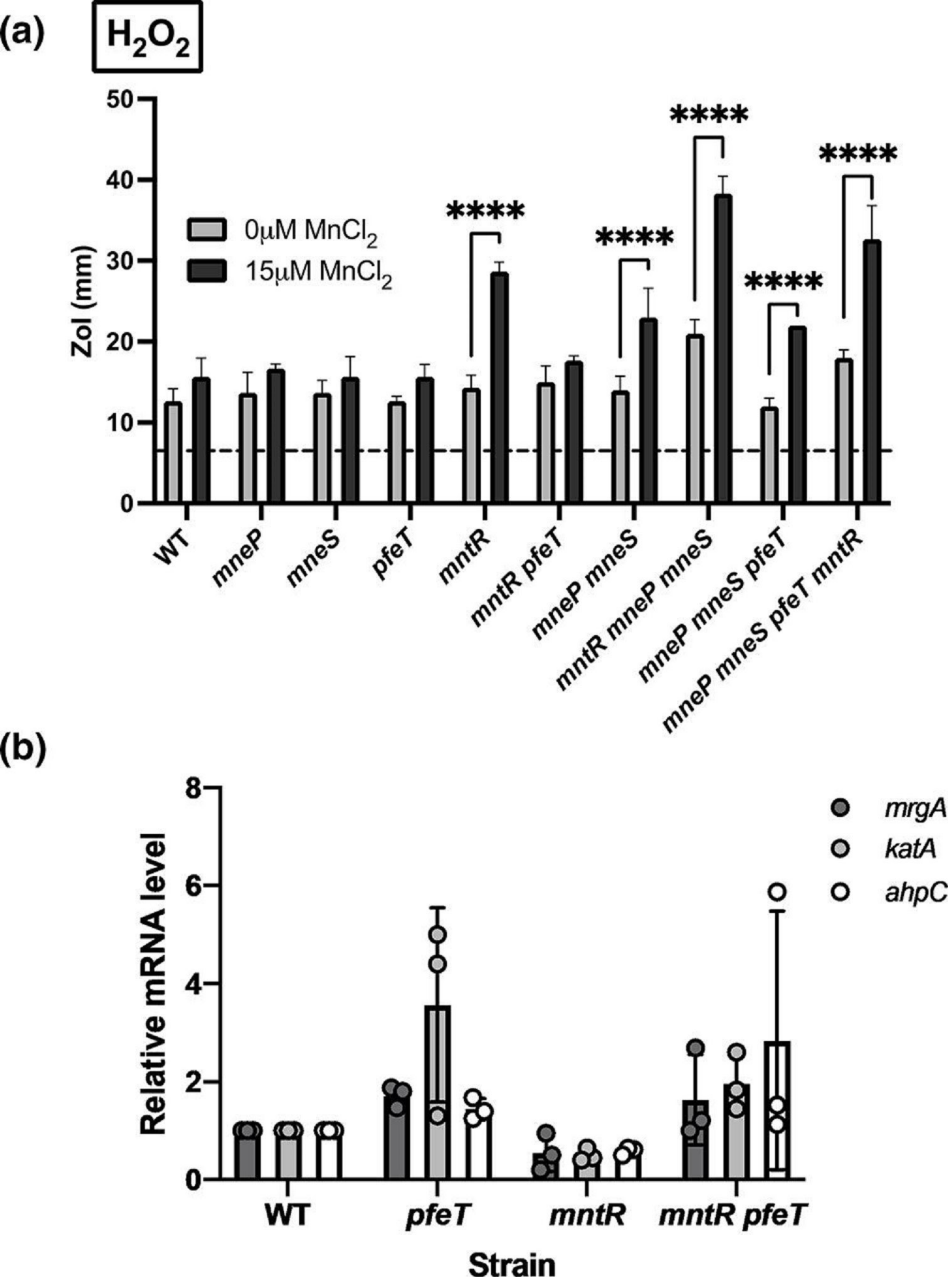
Loss of *mntR* exacerbates H_2_O_2_ sensitivity. (**a**) Mn^2+^ exacerbates peroxide sensitivity. H_2_O_2_ ZOI assays in LB plates with and without 15 µM MnCl_2_. Values shown are the mean ± sd (*n*=3). Black dashed line indicates diameter of the disc (6.5 mm). A two-way ANOVA test followed by a Šidák test for multiple comparisons was performed with corrections (****, <0.0001). (**b**) qRT-PCR data showing relative mRNA levels for *ahpC*, *katA* and *mrgA* genes measured in each strain background. Data obtained in mutant strains were normalized with respect to WT. Circles represent individual readings from each gene. Experiments were performed with three biological replicates and the means and standard deviations are shown.

Iron homeostasis and oxidative stress are linked, in part, through iron-dependent reduction of hydrogen peroxide (H_2_O_2_) to generate highly reactive hydroxyl radicals in Fenton reactions [46]. Cells that accumulate high levels of iron are often more susceptible to H_2_O_2_ stress [47, 48]. However, *pfeT* mutant cells were not more sensitive to H_2_O_2_ than WT (Fig. 6a), which is in line with past work showing this same phenotype [11].

In contrast with *pfeT* mutants, it has been reported that *mntR* mutants are more H_2_O_2_-sensitive than WT in LB medium [15, 49]. However, this phenotype is very sensitive to the precise metal content of the medium. In the present experiments, an *mntR* single mutant was not significantly more sensitive to H_2_O_2_ than WT in LB but was dramatically more sensitive in LB amended with 15 µM Mn^2+^ (Fig. 6a). This Mn^2+^-dependent increase in H_2_O_2_ sensitivity was not observed in the *mntR pfeT* double mutant. The effect of MntR on H_2_O_2_ sensitivity likely reflects both a decrease in *mneP* and *mneS* expression, and increased metal import. This is apparent since an *mntR mneP mneS* triple mutant is more H_2_O_2_-sensitive than the *mneP mneS* double mutant (Fig. 6a). In sum, inactivation of *pfeT* in the *mntR* mutant increases Fe^2+^ sensitivity (Fig. 1) but relieves Mn^2+^-dependent H_2_O_2_ sensitivity (Fig. 6a).

We hypothesized that these effects were likely due to metal-dependent changes in the activity of the PerR metalloregulator. PerR binds either Fe^2+^ or Mn^2+^ at its metal sensing site to affect repression. Upon H_2_O_2_ exposure, PerR : Fe undergoes metal-catalyzed oxidation [22, 23] and thereby derepresses peroxide resistance genes. In contrast, PerR : Mn is insensitive to H_2_O_2_ and remains an active repressor [22]. We measured *katA, ahpCF* and *mrgA* expression levels in WT, *pfeT*, *mntR* and *mntR pfeT* strains in LB to see how changes in Fe^2+^ and Mn^2+^ affect the PerR regulon. Cells lacking PfeT have higher mRNA levels of these H_2_O_2_ resistance genes, whereas in the *mntR* mutant expression was reduced (Fig. 6b). Thus, H_2_O_2_ resistance is affected by the relative levels of Fe^2+^ and Mn^2+^ as sensed by the PerR metalloregulator.

## Discussion

Bacteria must acquire essential metal ions from the environment, and this requires the regulated expression of high-affinity metal-uptake systems [2, 50]. To avoid metal ion intoxication, these high-affinity importers are counterbalanced by mechanisms for the sequestration or efflux of metals. As conditions change, the levels of the uptake and efflux transporters in the membrane can be modulated by metalloregulatory proteins [2, 51]. However, this is a relatively slow process and not suitable for controlling metal transport in response to rapid changes. A robust response to changing metal levels therefore likely requires a dynamic equilibrium in which cells sustain a potential for both uptake and efflux across a wide range of environmental metal levels.

In *B. subtilis *, iron and manganese homeostasis are intimately linked [1]. Iron efflux is promoted by the PfeT ATPase, which can be transcriptionally induced by either high iron or peroxide stress [10, 11]. Biochemical measurements suggest that iron efflux by PfeT is limited by a relatively low affinity for Fe^2+^, and that the efflux pump operates most effectively only under iron overload conditions [11]. The affinity of efflux ATPases for their cognate metal substrates likely varies depending on the organism, and the optimal concentration of the cytoplasmic labile metal ion pool. For example, induction of the *Listeria monocytogenes* PfeT orthologue, FrvA, in *B. subtilis* leads to a depletion of intracellular iron and induction of the Fur regulon [7], consistent with a higher measured affinity for iron as judged by ATPase activation [33]. Thus, one mechanism to optimize transporter activity is to appropriately tune substrate affinity. Alternatively, transport activity may be regulated allosterically. For example, the major Mg^2+^ importer MgtE is allosterically inhibited by Mg^2+^. However, allosteric mechanisms also come with risks, and elevated Mn^2+^ may inappropriately repress Mg^2+^ import [52].

**Fig. 7.**
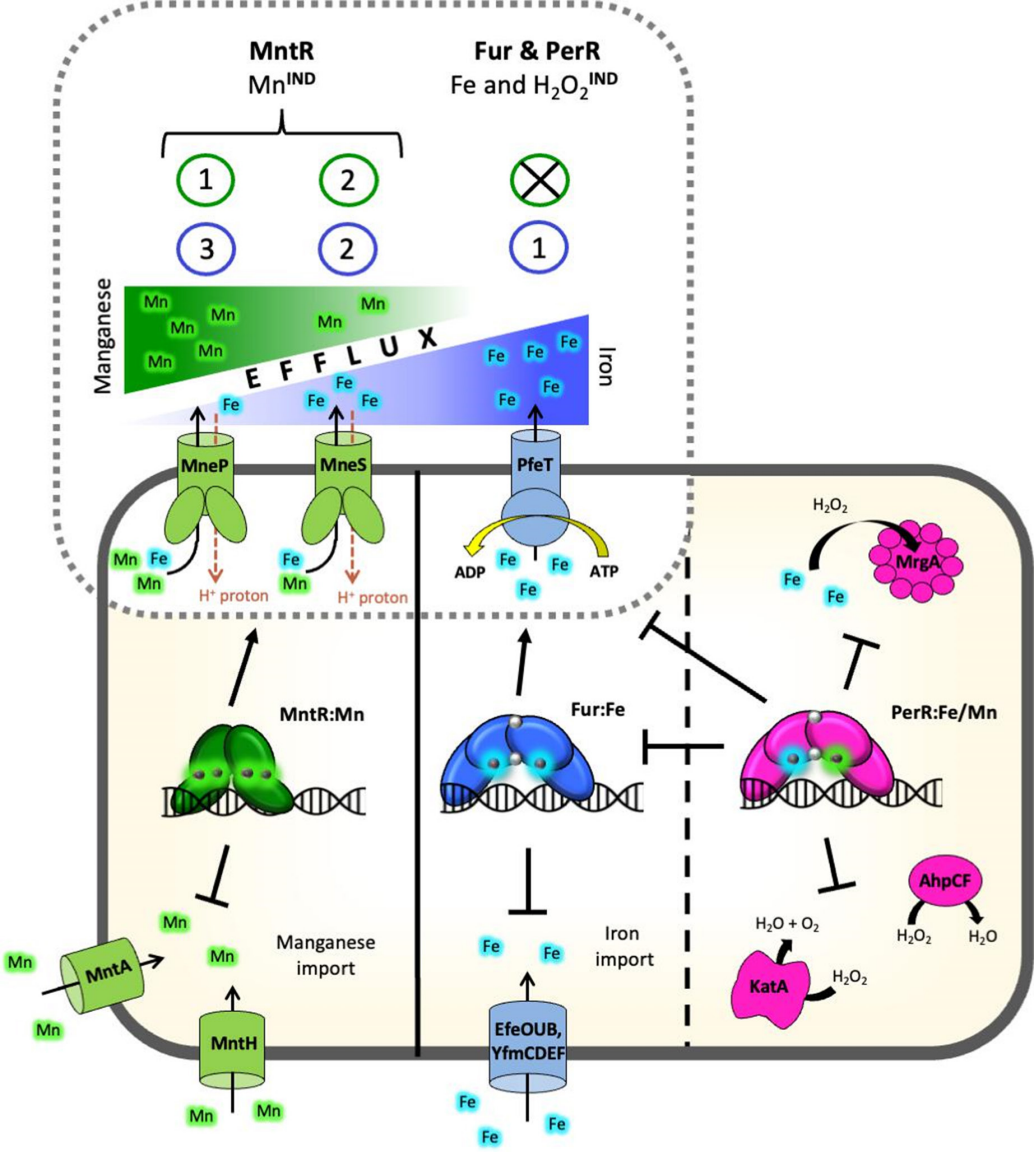
*
Bacillus subtilis* iron and manganese homeostasis and the oxidative stress response. Iron homeostasis in *B. subtilis* is controlled by Fur, which binds iron at its metal sensing site (Fur : Fe) to effect repression of iron acquisition systems and activate expression of the P_1B4_-type ATPase iron efflux pump PfeT. Both *fur* and *pfeT* are also part of the PerR regulon. PerR binds to either manganese or iron at its metal-sensing site, but only PerR : Fe reacts with H_2_O_2_ to allow expression of the detoxification enzymes catalase (KatA) and alkyl hydroperoxide reductase (AhpCF) and the iron sequestering mini-ferritin MrgA. Independently of these two regulons, manganese homeostasis is regulated by MntR, which represses expression of two manganese import systems (MntH and MntABCD) and activates expression of MneP and MneS. The primary efflux pump in excess iron conditions is PfeT (shown here by the blue number 1 circle) and it is induced (^IND^) by both Fe^2+^ and H_2_O_2_. MneP and MneS are the main manganese efflux pumps and are induced by Mn via MntR. MneP is the primary manganese exporter (green number 1), whereas MneS plays a secondary role (green number 2). The results herein suggest that this order may be reversed with respect to Fe^2+^ efflux. The dashed black line shows regulon overlap, and the solid black line separates distinct, non-overlapping regulons.

Here, we have revealed a role for the MneP and MneS CDF proteins in iron homeostasis. Expression of MneP and MneS is controlled by MntR, and the synthesis of both proteins is induced by Mn^2+^ [19] but not by Fe^2+^ (Fig. S1). Since expression of *mneP* and *mneS* is largely dependent on MntR, *mntR* mutant strains are highly sensitive to Mn^2+^ [19]. However, *mneP mneS* mutants are also sensitive to iron (Fig. 2) and have increased intracellular iron as monitored using a bioreporter (Fig. 5). This implies that, even at their basal level of expression, MneP and MneS play a role in helping cells balance their intracellular iron pools. Fe^2+^ efflux in *B. subtilis* was previously attributed to the PerR and Fur controlled P_1B4_-type ATPase, PfeT [11]. In light of our current results, we suggest a model wherein MneP and MneS act as a ‘release valve’ for low-level iron fluctuations until a drastic increase in iron levels induces PfeT (Fig. 7).

CDF proteins are ubiquitous dimeric transporters that harness energy from the proton motive force for metal cation export. Each monomer contains an N-terminal six transmembrane domain (TMD) and a variable C-terminal cytoplasmic domain (CTD) [53, 54]. Structural studies of the *Escherichia coli* CDF efflux pump FieF (YiiP) have provided insight into how metal export occurs [55, 56]. Each monomer has four putative metal binding sites (A, B, C1 and C2) and it has been proposed that metal selectivity is dependent on binding site A at the interface of transmembrane helices 2 and 5 [55–57]. These conserved Mn^2+^ (DD–DD) and Fe^2+^ (HD–HD) motifs are correlated with metal specificity [57]. MneP (HD–DD) and MneS (ND–DD) contain ‘hybrid’ motifs that may thereby enable a broader selectivity. Other CDF proteins have also been reported to have dual Fe^2+^/Mn^2+^ selectivity. In *Rhizobium etli *, mutants lacking EmfA are Mn^2+^-sensitive and *emfA* shows Mn^2+^-dependent induction, however in *E. coli* EmfA confers a modest level of iron resistance [58]. Similarly, although *Enterococcus faecalis* MntE is specifically induced by Mn^2+^, an *mntE* mutant accumulates both Fe^2+^ and Mn^2+^ [59]. Further, the *Salmonella enterica* FieF protein can mediate efflux of both Fe^2+^ and Mn^2+^ and at least *in vitro* also functions with Zn^2+^ [60].

In addition to affecting iron homeostasis, dysregulation of manganese homeostasis in an *mntR* null mutant affects H_2_O_2_ sensitivity. Manganese is often considered to be an antioxidant that alleviates oxidative stress through metalation of Mn^2+^-utilizing superoxide dismutase (SOD) and oxidized iron mono-nuclear enzymes [46, 61–63]. However, Mn^2+^ sensitizes *B. subtilis* to H_2_O_2_ toxicity [15, 20]. This can be rationalized due to the ability of Mn^2+^ to tightly repress the PerR regulon [1]. Indeed, this same mechanism accounts for the ability of mutations in the *mntE* Mn^2+^ efflux pump to sensitize *Streptococcus pyogenes* to H_2_O_2_ killing [64]. Loss of *mntE* also increases sensitivity to the superoxide-generating compound paraquat in *Staphylococus aureus* [65]. Conversely, in *Streptococcus pneumonia* an *mntE* mutant appears to be more resistant to oxidative stress [66]. One common theme, however, is that fitness in the host environment, and therefore virulence, is heavily reliant on the ability of bacteria to both compete with the host for essential metal ions, and to balance high-affinity import with specific efflux systems [50, 63, 67–69].

## Supplementary Data

Supplementary material 1

## References

[1] Helmann JD (2014). Specificity of metal sensing: iron and manganese homeostasis in *Bacillus subtilis*. J Biol Chem.

[2] Chandrangsu P, Rensing C, Helmann JD (2017). Metal homeostasis and resistance in bacteria. Nat Rev Microbiol.

[3] Sarvan S, Butcher J, Stintzi A, Couture JF (2018). Variation on a theme: investigating the structural repertoires used by ferric uptake regulators to control gene expression. Biometals.

[4] Sevilla E, Bes MT, Peleato ML, Fillat MF (2021). Fur-like proteins: Beyond the ferric uptake regulator (Fur) paralog. Arch Biochem Biophys.

[5] Bsat N, Herbig A, Casillas-Martinez L, Setlow P, Helmann JD (1998). *Bacillus subtilis* contains multiple Fur homologues: identification of the iron uptake (Fur) and peroxide regulon (PerR) repressors. Mol Microbiol.

[6] Ollinger J, Song KB, Antelmann H, Hecker M, Helmann JD (2006). Role of the Fur regulon in iron transport in *Bacillus subtilis*. J Bacteriol.

[7] Pi H, Helmann JD (2017). Sequential induction of Fur-regulated genes in response to iron limitation in *Bacillus subtilis*. Proc Natl Acad Sci U S A.

[8] Gaballa A, Antelmann H, Aguilar C, Khakh SK, Song K-B (2008). The *Bacillus subtilis* iron-sparing response is mediated by a Fur-regulated small RNA and three small, basic proteins. Proc Natl Acad Sci U S A.

[9] Smaldone GT, Antelmann H, Gaballa A, Helmann JD (2012). The FsrA sRNA and FbpB protein mediate the iron-dependent induction of the *Bacillus subtilis* lutABC iron-sulfur-containing oxidases. J Bacteriol.

[10] Pinochet-Barros A, Helmann JD (2020). *Bacillus subtilis* Fur Is a transcriptional activator for the PerR-repressed *pfeT* Gene, encoding an iron efflux pump. J Bacteriol.

[11] Guan G, Pinochet-Barros A, Gaballa A, Patel SJ, Argüello JM (2015). PfeT, a P1B4 -type ATPase, effluxes ferrous iron and protects *Bacillus subtilis* against iron intoxication. Mol Microbiol.

[12] Pi H, Helmann JD (2017). Ferrous iron efflux systems in bacteria. Metallomics.

[13] Brown JB, Lee MA, Smith AT (2021). Ins and Outs: Recent advancements in membrane protein-mediated prokaryotic ferrous iron transport. Biochemistry.

[14] Oh YK, Freese E (1976). Manganese requirement of phosphoglycerate phosphomutase and its consequences for growth and sporulation of *Bacillus subtilis*. J Bacteriol.

[15] Que Q, Helmann JD (2000). Manganese homeostasis in *Bacillus subtilis* is regulated by MntR, a bifunctional regulator related to the diphtheria toxin repressor family of proteins. Mol Microbiol.

[16] Glasfeld A, Guedon E, Helmann JD, Brennan RG (2003). Structure of the manganese-bound manganese transport regulator of *Bacillus subtilis*. Nat Struct Biol.

[17] McGuire AM, Cuthbert BJ, Ma Z, Grauer-Gray KD, Brunjes Brophy M (2013). Roles of the A and C sites in the manganese-specific activation of MntR. Biochemistry.

[18] Guedon E, Moore CM, Que Q, Wang T, Ye RW (2003). The global transcriptional response of *Bacillus subtilis* to manganese involves the MntR, Fur, TnrA and sigmaB regulons. Mol Microbiol.

[19] Huang X, Shin JH, Pinochet-Barros A, Su TT, Helmann JD (2017). *Bacillus subtilis* MntR coordinates the transcriptional regulation of manganese uptake and efflux systems. Mol Microbiol.

[20] Chen L, Keramati L, Helmann JD (1995). Coordinate regulation of *Bacillus subtilis* peroxide stress genes by hydrogen peroxide and metal ions. Proc Natl Acad Sci U S A.

[21] Herbig AF, Helmann JD (2001). Roles of metal ions and hydrogen peroxide in modulating the interaction of the *Bacillus subtilis* PerR peroxide regulon repressor with operator DNA. Mol Microbiol.

[22] Lee JW, Helmann JD (2006). The PerR transcription factor senses H₂O₂ by metal-catalysed histidine oxidation. Nature.

[23] Jacquamet L, Traoré DAK, Ferrer J-L, Proux O, Testemale D (2009). Structural characterization of the active form of PerR: insights into the metal-induced activation of PerR and Fur proteins for DNA binding. Mol Microbiol.

[24] Traoré DAK, El Ghazouani A, Jacquamet L, Borel F, Ferrer J-L (2009). Structural and functional characterization of 2-oxo-histidine in oxidized PerR protein. Nat Chem Biol.

[25] Ahn BE, Baker TA (2016). Oxidization without substrate unfolding triggers proteolysis of the peroxide-sensor, PerR. Proc Natl Acad Sci U S A.

[26] Pinochet-Barros A, Helmann JD (2018). Redox sensing by Fe²⁺ in bacterial fur family metalloregulators. Antioxid Redox Signal.

[27] Ma Z, Lee JW, Helmann JD (2011). Identification of altered function alleles that affect *Bacillus subtilis* PerR metal ion selectivity. Nucleic Acids Res.

[28] Ji C-J, Kim J-H, Won Y-B, Lee Y-E, Choi T-W (2015). *Staphylococcus aureus* PerR Is a hypersensitive hydrogen peroxide sensor using iron-mediated histidine oxidation. J Biol Chem.

[29] Helmann JD, Wu MFW, Gaballa A, Kobel PA, Morshedi MM (2003). The global transcriptional response of *Bacillus subtilis* to peroxide stress is coordinated by three transcription factors. J Bacteriol.

[30] Faulkner MJ, Helmann JD (2011). Peroxide stress elicits adaptive changes in bacterial metal ion homeostasis. Antioxid Redox Signal.

[31] Faulkner MJ, Ma Z, Fuangthong M, Helmann JD (2012). Derepression of the *Bacillus subtilis* PerR peroxide stress response leads to iron deficiency. J Bacteriol.

[32] Chen L, Helmann JD (1995). *Bacillus subtilis* MrgA is a Dps(PexB) homologue: evidence for metalloregulation of an oxidative-stress gene. Mol Microbiol.

[33] Pi H, Patel SJ, Argüello JM, Helmann JD (2016). The Listeria monocytogenes Fur-regulated virulence protein FrvA is an Fe(II) efflux P1B4 -type ATPase. Mol Microbiol.

[34] VanderWal AR, Makthal N, Pinochet-Barros A, Helmann JD, Olsen RJ (2017). Iron efflux by PmtA is critical for oxidative stress resistance and contributes significantly to group a *Streptococcus* virulence. Infect Immun.

[35] Patel SJ, Lewis BE, Long JE, Nambi S, Sassetti CM (2016). Fine-tuning of substrate affinity leads to alternative roles of *Mycobacterium tuberculosis* Fe^2+^ -ATPases. J Biol Chem.

[36] Turner AG, Djoko KY, Ong C-LY, Barnett TC, Walker MJ (2019). Group A *Streptococcus* co-ordinates manganese import and iron efflux in response to hydrogen peroxide stress. Biochem J.

[37] Turner AG, Ong C-LY, Djoko KY, West NP, Davies MR (2017). The PerR-Regulated P_1B-4_-Type ATPase (PmtA) acts as a ferrous iron efflux pump in *Streptococcus pyogenes*. Infect Immun.

[38] Slack FJ, Mueller JP, Sonenshein AL (1993). Mutations that relieve nutritional repression of the *Bacillus subtilis* dipeptide permease operon. J Bacteriol.

[39] Quisel JD, Burkholder WF, Grossman AD (2001). In vivo effects of sporulation kinases on mutant Spo0A proteins in *Bacillus subtilis*. J Bacteriol.

[40] Davies BW, Walker GC (2007). Disruption of sitA compromises *Sinorhizobium meliloti* for manganese uptake required for protection against oxidative stress. J Bacteriol.

[41] Fleming MD, Trenor CC, Su MA, Foernzler D, Beier DR (1997). *Microcytic anaemia* mice have a mutation in Nramp2, a candidate iron transporter gene. Nat Genet.

[42] Gunshin H, Mackenzie B, Berger UV, Gunshin Y, Romero MF (1997). Cloning and characterization of a mammalian proton-coupled metal-ion transporter. Nature.

[43] Runyen-Janecky L, Dazenski E, Hawkins S, Warner L (2006). Role and regulation of the Shigella flexneri sit and MntH systems. Infect Immun.

[44] Sabri M, Léveillé S, Dozois CM (2006). A SitABCD homologue from an avian pathogenic *Escherichia coli* strain mediates transport of iron and manganese and resistance to hydrogen peroxide. Microbiology.

[45] Yeowell HN, White JR (1982). Iron requirement in the bactericidal mechanism of streptonigrin. Antimicrob Agents Chemother.

[46] Imlay JA (2013). The molecular mechanisms and physiological consequences of oxidative stress: lessons from a model bacterium. Nat Rev Microbiol.

[47] Frawley ER, Fang FC (2014). The ins and outs of bacterial iron metabolism. Mol Microbiol.

[48] Khademian M, Imlay JA (2021). How microbes evolved to tolerate oxygen. Trends Microbiol.

[49] Randazzo P, Anba-Mondoloni J, Aubert-Frambourg A, Guillot A, Pechoux C (2020). *Bacillus subtilis* regulators MntR and Zur participate in redox cycling, antibiotic sensitivity, and cell wall plasticity. J Bacteriol.

[50] Jordan MR, Wang J, Capdevila DA, Giedroc DP (2020). Multi-metal nutrient restriction and crosstalk in metallostasis systems in microbial pathogens. Curr Opin Microbiol.

[51] Osman D, Martini MA, Foster AW, Chen J, Scott AJP (2019). Bacterial sensors define intracellular free energies for correct enzyme metalation. Nat Chem Biol.

[52] Hohle TH, O’Brian MR (2014). Magnesium-dependent processes are targets of bacterial manganese toxicity. Mol Microbiol.

[53] Kolaj-Robin O, Russell D, Hayes KA, Pembroke JT, Soulimane T (2015). Cation Diffusion Facilitator family: Structure and function. FEBS Lett.

[54] Montanini B, Blaudez D, Jeandroz S, Sanders D, Chalot M (2007). Phylogenetic and functional analysis of the Cation Diffusion Facilitator (CDF) family: improved signature and prediction of substrate specificity. BMC Genomics.

[55] Lu M, Chai J, Fu D (2009). Structural basis for autoregulation of the zinc transporter YiiP. Nat Struct Mol Biol.

[56] Lu M, Fu D (2007). Structure of the zinc transporter YiiP. Science.

[57] Martin JE, Giedroc DP (2016). Functional determinants of metal ion transport and selectivity in paralogous cation diffusion facilitator transporters CzcD and MntE in *Streptococcus pneumoniae*. J Bacteriol.

[58] Cubillas C, Vinuesa P, Tabche ML, Dávalos A, Vázquez A (2014). The cation diffusion facilitator protein EmfA of *Rhizobium etli* belongs to a novel subfamily of Mn(2+)/Fe(2+) transporters conserved in α-proteobacteria. Metallomics.

[59] Lam LN, Wong JJ, Chong KKL, Kline KA (2020). *Enterococcus faecalis* manganese exporter MntE alleviates manganese toxicity and is required for mouse gastrointestinal colonization. Infect Immun.

[60] Ouyang A, Gasner KM, Neville SL, McDevitt CA, Frawley ER (2022). MntP and YiiP contribute to manganese efflux in *Salmonella enterica* serovar typhimurium under conditions of manganese overload and nitrosative stress. Microbiol Spectr.

[61] Anjem A, Imlay JA (2012). Mononuclear iron enzymes are primary targets of hydrogen peroxide stress. J Biol Chem.

[62] Sobota JM, Gu M, Imlay JA (2014). Intracellular hydrogen peroxide and superoxide poison 3-deoxy-D-arabinoheptulosonate 7-phosphate synthase, the first committed enzyme in the aromatic biosynthetic pathway of *Escherichia coli*. J Bacteriol.

[63] Juttukonda LJ, Skaar EP (2015). Manganese homeostasis and utilization in pathogenic bacteria. Mol Microbiol.

[64] Turner AG, Ong C-L, Gillen CM, Davies MR, West NP (2015). Manganese homeostasis in group A Streptococcus is critical for resistance to oxidative stress and virulence. mBio.

[65] Grunenwald CM, Choby JE, Juttukonda LJ, Beavers WN, Weiss A (2019). Manganese detoxification by MntE is critical for resistance to oxidative stress and virulence of *Staphylococcus aureus*. mBio.

[66] Rosch JW, Gao G, Ridout G, Wang YD, Tuomanen EI (2009). Role of the manganese efflux system mntE for signalling and pathogenesis in *Streptococcus pneumoniae*. Mol Microbiol.

[67] Begg SL (2019). The role of metal ions in the virulence and viability of bacterial pathogens. Biochem Soc Trans.

[68] Becker KW, Skaar EP (2014). Metal limitation and toxicity at the interface between host and pathogen. FEMS Microbiol Rev.

[69] Lopez CA, Skaar EP (2018). The impact of dietary transition metals on host-bacterial interactions. Cell Host Microbe.

