# Supplementary material for: Iron homeostasis in Bacillus subtilis relies on three differentially expressed efflux systems
Source: Microbiology (Reading). 2023 Jan 17;169(1):001289. doi: 10.1099/mic.0.001289 (PMC9993123; doi:10.1099/mic.0.001289)
Supplement: Supplementary material 1 [file mic-169-1289-s001.pdf]

Supplementary Material

Iron Homeostasis in *Bacillus subtilis* Relies on Three Differentially Expressed Efflux Systems

Caroline H. Steingard, Azul Pinochet-Barros, Brian M. Wendel, & John D. Helmann

Table S1. Strains and plasmids used in this study

Table S2. Oligonucleotides used in this study

Fig. S1. *mneP* and *mneS* are not induced by iron

Fig. S2. Fe^{2+} sensitivity of Mn Importer deletion strains

Fig. S3. Streptonigrin sensitivity of WT, *pfeT, mntR,* double mutants and PfeT complemented strain.

Fig. S4. Streptonigrin sensitivity to monitor MneP and MneS complementation (data in Fig. 3B)

Table S1) Strains and plasmids used in this study

STRAIN	GENOTYPE	REFERENCE
CU1065	W168 attSPβ trpC2	Laboratory stock
ZB307A	W168 SPβ c2Δ2::Tn917::pSK10Δ6	Laboratory stock
HB18022	CU1065 SPβ c2Δ2::Tn917::ϕ(pfeT-cat-lacZ)	(Pinochet-Barros & Helmann 2020)
HB8116	CU1065 pfeT::kan SPβ c2Δ2::Tn917::ϕ(pfeT-cat-lacZ)	Laboratory stock
HB17802	CU1065 pfeT::spc	(Guan et al. 2015)
HB2621	CU1065 mntR::kan	(Guedon et al. 2003)
HB17806	CU1065 mntR::kan pfeT::spc	(Guan et al. 2015)
HB18190	CU1065 mntR::kan pfeT::spc amyE::Pspac-pfeT (cat)	This work
HB17767	CU1065 ΔmntA	(Huang et al. 2017)
HB17768	CU1065 ΔmntH	(Huang et al. 2017)
HB17780	CU1065 mntR::tet ΔmntH	(Huang et al. 2017)
HB17779	CU1065 mntR::tet ΔmntA	(Huang et al. 2017)
HB17827	CU1065 pfeT::spc mntH::mls	(Guan et al. 2015)
HB17828	CU1065 mntR::kan pfeT::spc mntH::mls	(Guan et al. 2015)
HB17773	CU1065 ΔmneP	(Huang et al. 2017)
HB17769	CU1065 ΔmneS	(Huang et al. 2017)
HB17784	CU1065 ΔmneP ΔmneS	(Huang et al. 2017)
HB17795	CU1065 mntR::tet ΔmneP ΔmneS	(Huang et al. 2017)
HB17796	CU1065 thrC::P_{mneS}-lacZ (mls)	(Huang et al. 2017)
HB17797	CU1065 thrC::P_{mneP}-lacZ (mls)	(Huang et al. 2017)
HB18195	CU1065 pfeT::spc ΔmneP	This work
HB18194	CU1065 pfeT::spc ΔmneS	This work
HB19554	CU1065 pfeT::spc ΔmneP ΔmneS	(Huang et al. 2017)
HB18196	CU1065 ΔmneP ΔmneS pfeT::spc mntR::kan	This work
HB26501	CU1065 ΔmneP SPβ c2Δ2::Tn917::ϕ(pfeT-cat-lacZ)	This work
HB26502	CU1065 ΔmneS SPβ c2Δ2::Tn917::ϕ(pfeT-cat-lacZ)	This work
HB18198	CU1065 Pspac-mneP (cat)	This work
HB18199	CU1065 Pspac-mneS (cat)	This work
HB18202	CU1065 pfeT::spc Pspac-mneP (cat)	This work
HB18203	CU1065 pfeT::spc Pspac-mneS (cat)	This work
HB18210	CU1065 mntR::kan Pspac-mneP (cat)	This work
HB18211	CU1065 mntR::kan Pspac-mneS (cat)	This work
HB18200	CU1065 mntR::kan pfeT::spc Pspac-mneP (cat)	This work
HB18201	CU1065 mntR::kan pfeT::spc Pspac-mneS (cat)	This work
HB18204	CU1065 Pspac-mntH (cat)	This work
HB18205	CU1065 pfeT::spc Pspac-mntH (cat)	This work
HB18206	CU1065 mntR::kan Pspac-mntH (cat)	This work
HB18207	CU1065 mntR::kan pfeT::spc Pspac-mntH (cat)	This work
HB18217	CU1065 ΔmneP ΔmneS SPβ c2Δ2::Tn917::ϕ(pfeT-cat-lacZ)	This work
HB18218	CU1065 pfeT::spc ΔmneP ΔmneS SPβ c2Δ2::Tn917::ϕ(pfeT-cat-lacZ)	This work
**PLASMID**	**DESCRIPTION**	**REFERENCE**
pJPM122	cat-lacZ operon fusion vector for SPβ.	(Slack et al. 1993)
pPL82	Expression of gene under P_{spac} promoter.	(Quisel et al. 2001)

References

- Guan, G., Pinochet-Barros, A., Gaballa, A., Patel, S. J., Arguello, J. M., & Helmann, J. D. (2015). PfeT, a P1B4 -type ATPase, effluxes ferrous iron and protects *Bacillus subtilis* against iron intoxication. *Mol Microbiol*, *98*(4), 787-803. https://doi.org/10.1111/mmi.13158
- Guedon, E., & Helmann, J. D. (2003). Origins of metal ion selectivity in the DtxR/MntR family of metalloregulators. *Mol Microbiol*, *48*(2), 495-506. https://doi.org/10.1046/j.1365-2958.2003.03445.x
- Huang, X., Shin, J. H., Pinochet-Barros, A., Su, T. T., & Helmann, J. D. (2017). *Bacillus subtilis* MntR coordinates the transcriptional regulation of manganese uptake and efflux systems. *Mol Microbiol*, *103*(2), 253-268. https://doi.org/10.1111/mmi.13554
- Pinochet-Barros, A., & Helmann, J. D. (2020*). Bacillus subtilis* Fur Is a Transcriptional Activator for the PerR-Repressed pfeT Gene, Encoding an Iron Efflux Pump. *J Bacteriol*, *202*(8). https://doi.org/10.1128/JB.00697-19
- Quisel, J. D., Burkholder, W. F., & Grossman, A. D. (2001). In vivo effects of sporulation kinases on mutant Spo0A proteins in *Bacillus subtilis*. *J Bacteriol*, *183*(22), 6573-6578. https://doi.org/10.1128/JB.183.22.6573-6578.2001
- Slack, F. J., Mueller, J. P., & Sonenshein, A. L. (1993). Mutations that relieve nutritional repression of *the Bacillus subtilis* dipeptide permease operon. *J Bacteriol*, *175*(15), 4605-4614. https://doi.org/10.1128/jb.175.15.4605-4614.1993

Table S2) Oligonucleotides used in this study

NUMBER	NAME	SEQUENCE
7750	qPCR mrgA-F	GCTCCACCGTTTCCATTGGTATGTGAA
7751	qPCR mrgA-R	CGTCTGTGATAGATGCATGCTCAGTGTA
8541	qPCR katA-F	CCTGCGACACTTCGCCACAT
8542	qPCR katA-R	GGTAATCAGGGTTTTCACCGGCAA
8543	qPCR ahpC-F	CCCAACTGAGCTTGAAGATCTTCAAGAACAA
8544	qPCR ahpC-R	CGTCAAGAACATCGAAGTTGCGAGAGAT
7277	bgal-pfeT-F	GCCAAGCTTCCCAACATCATTTTTGCTGAAT
7278	bgal-pfeT-R	GCGGATCCGGGTCGCGTTGAACGATAA
6416	mneS up F	CCTACAAATAGGCCGGCTCC
6417	mneS down R	TGGCGATGTCTCGTTTTCCA
6427	mneP up F	TCGCTGATCTTTCCGACCAA
6428	mneP down R	GCGTCGGGAGATCTTTCGTT
6420	mntH up F	CCGGCTTTGCTATTTTCCCG
6421	mntH down R	AGAACCGACCAAGAAGGTGC
8537	mneP-F	GGAACAGCAAAGCGATTATAGCGATTGCGTA
8538	mneP-R	GCACATCAACAGACGGCCTGATTA
8539	mneS-F	CCTGATGCTGATCGTTTACCGGTACAAT
8540	mneS-R	CGATCACAAACGCAGTGACCGTATCAAT
8533	mntH-F	GGCACCGACAGTGTGCTTCTT
8534	mntH-R	GCATTAATAGCCCCTGCAATCAGCAT
535a	pJPM122 check F	GTACATATTGTCGTTAGAACGCGGC
366	pJPM122 check R	ACTCTCCGTCGCTATTGTAACCAG
1293	cat-fwd	CGGCAATAGTTACCCTTATTATCAAG
1294	cat-rev	CCAGCGTGGACCGGCGAGGCTAGTTACCC

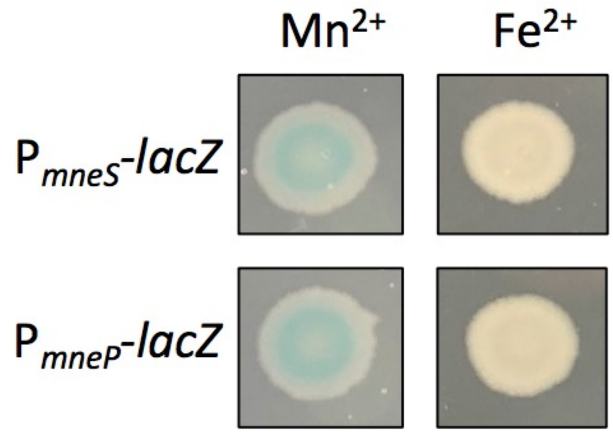

Fig. S1 ***mneP*** **and** ***mneS*** **are not induced by iron.**
Spot plating of WT cells containing transcriptional *lacZ* fusions of *mneP* and *mneS* onto LB plates containing X-Gal and either 250 µM $MnCl_2$ or 300 µM $FeSO_4$. Data is representative of more than three biological replicates.

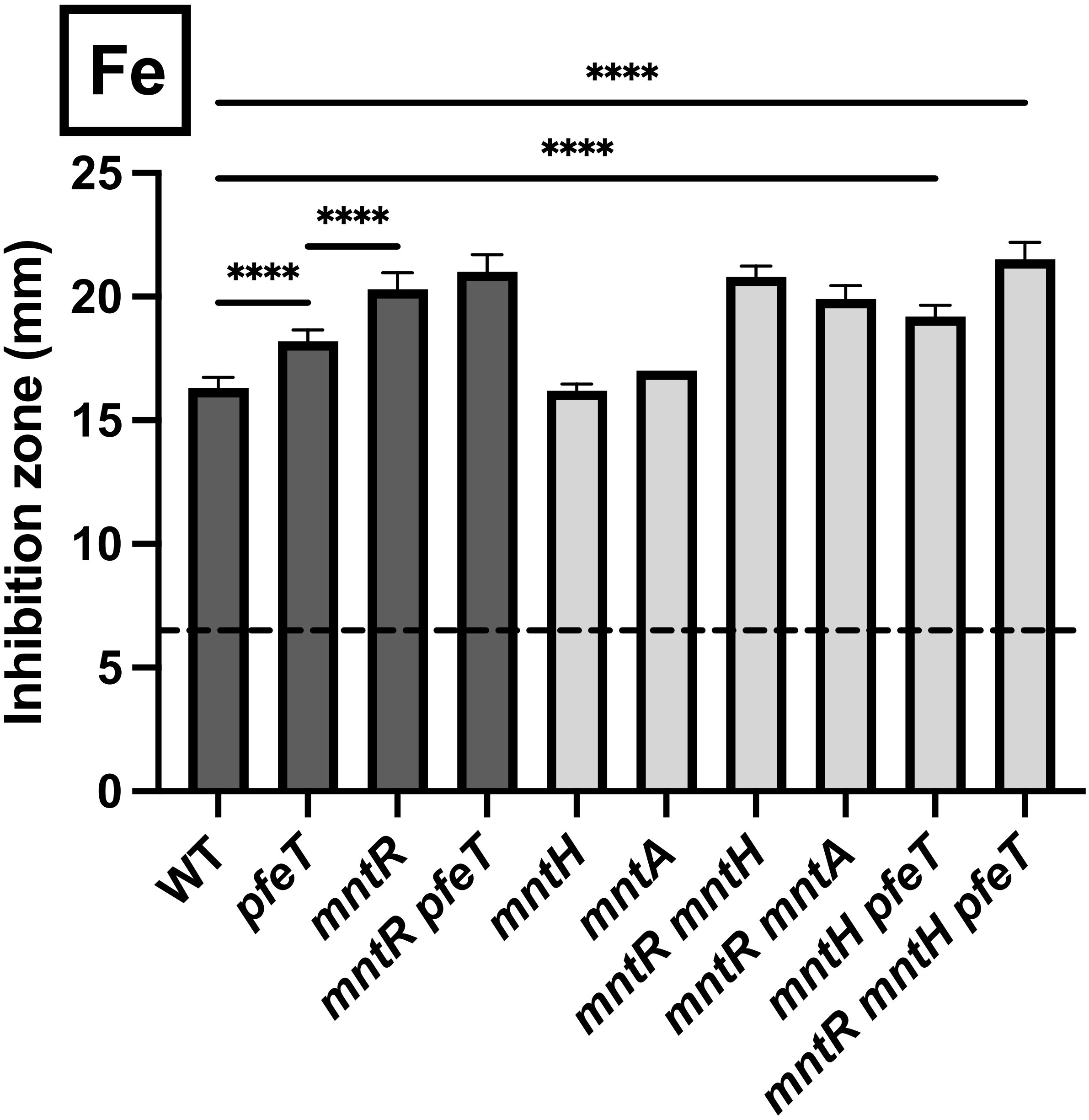

Fig. S2 Sensitivity of Mn Importer deletion strains to Fe^{2+} as monitored using a disk diffusion (zone of inhibition; ZoI) assays. Bars in dark gray show the control strains, which are the same data as in Fig. 1A. Bars shaded in light gray denote manganese import mutant strains. The results are expressed as the diameter ZoI (mm) and are shown as mean ± SD (n=5). Black dashed line indicates diameter of the disk (6.5 mm). A one-way ANOVA test with multiple comparison analysis was performed with a Tukey correction. Four asterisks represent a p-value of less than 0.0001.

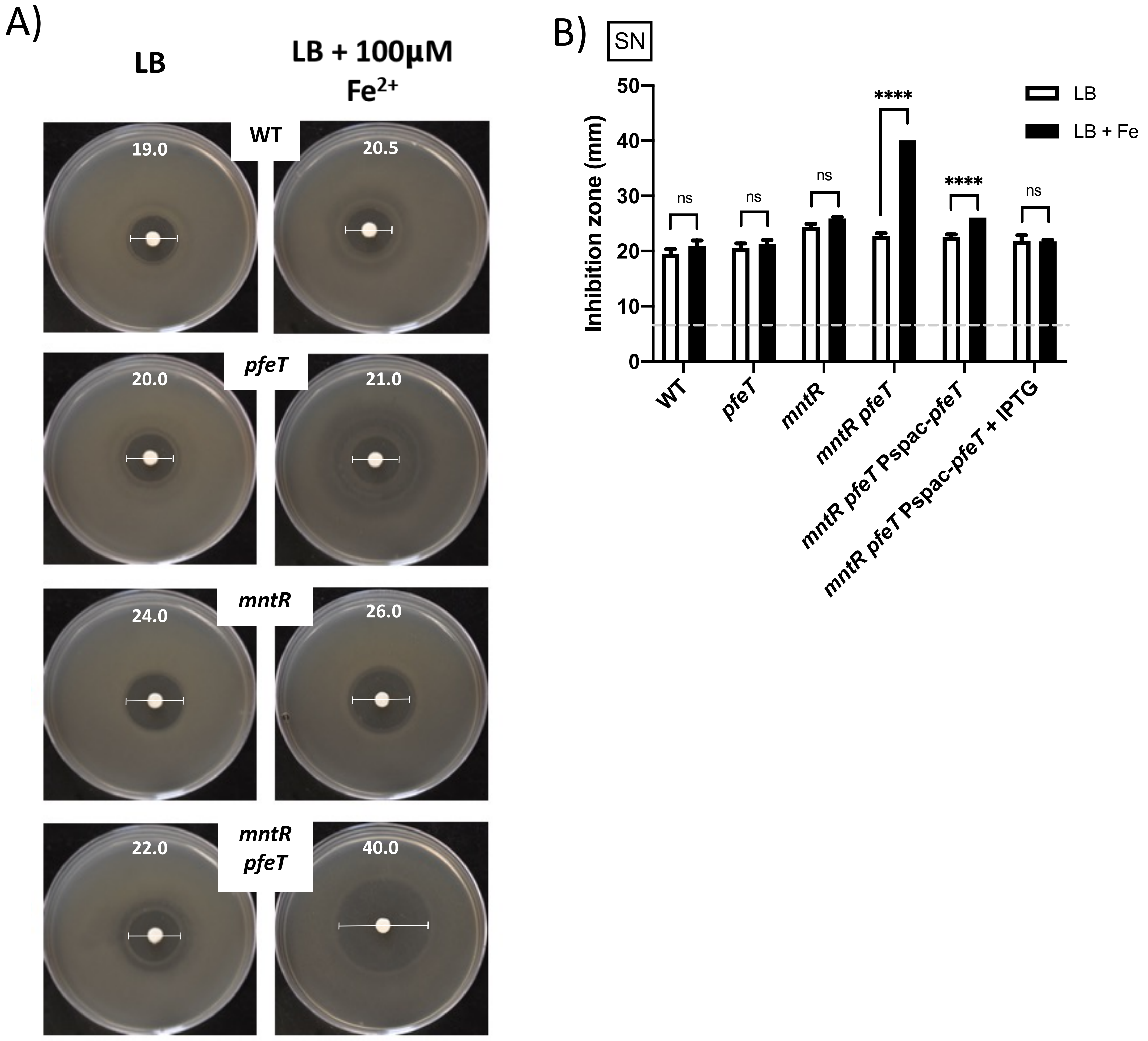

Fig. S3 Streptonigrin sensitivity of WT, *pfeT, mntR,* double mutants and PfeT complemented strain. A) Representative zone of inhibition plates for WT, *pfeT, mntR*, and double mutants in response to streptonigrin (SN) on LB with (right) or without (left) 100uM iron. **B)** Streptonigrin (SN) zone of inhibition assay in LB media with and without 100 µM Fe^{2+}. Light grey line indicates diameter of the disk (6.5 mm). The results are expressed as the diameter ZoI (mm) and are shown as mean ± SD (n=3). A two-way ANOVA test with multiple comparison was performed with a Šidák correction (**** = <0.0001; ns = no significance).

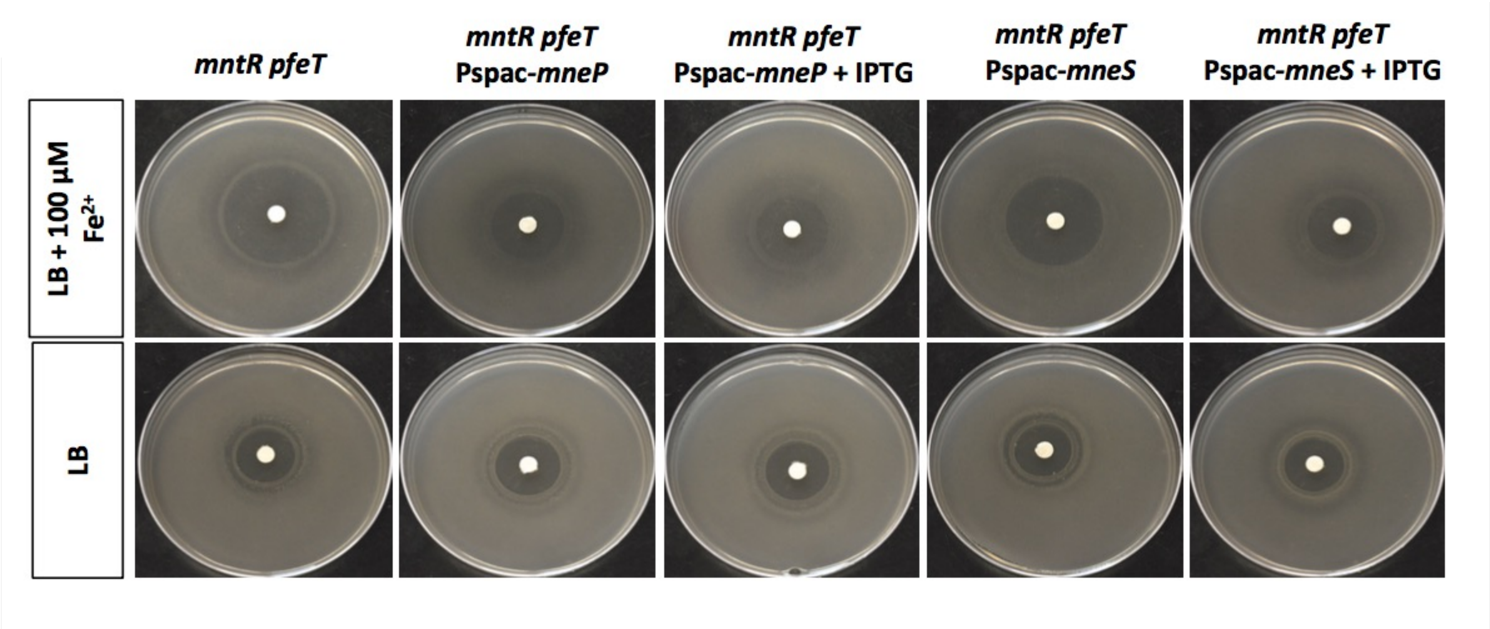

Fig. S4 Streptonigrin sensitivity to monitor MneP and MneS complementation (data in Fig. 3B). Images of streptonigrin (SN) zone of inhibition plates in LB media with and without 100 µM Fe on strains expressing *mneP* and *mneS* through an IPTG inducible promoter.
